# The Renal Composite Benefit of Sodium Glucose Co-Transporter 2 Inhibitors Should Ideally Be Assessed Based on a Standardised Definition: A Meta-Analysis of Randomised Controlled Trials

**DOI:** 10.3390/jcm12206462

**Published:** 2023-10-11

**Authors:** Samit Ghosal, Shamita Ghosal, Anuradha Ghosal

**Affiliations:** 1 Department of Medicine, Nightingale Hospital, Kolkata 700071, India; 2 Department of Obstetrics and Gynaecology, Nightingale Hospital, Kolkata 700071, India; shamita_07@yahoo.co.in; 3 School of Medicine, Aston University, Birmingham B4 7ET, UK; anuradhaghosal097@gmail.com

**Keywords:** SGLT-2is, renal composite, eGFR decline, meta-analysis, prediction interval, uniform definition

## Abstract

(1) Background: Chronic kidney disease (CKD) is extremely common against the backdrop of type 2 diabetes (T2D), accounting for nearly 30–40% of cases. The conventional management strategy relie predominantly on metabolic control and the renin–angiotensin–aldosterone system (RAAS) blockage. In the last decade, sodium glucose cotransporter 2 inhibitors (SGLT-2is) have emerged as the leading molecules preventing the development of, as well as retarding, the progression to CKD. Although the evidence in support of SGLT-2is is overwhelming, the definition of renal composite outcome in the trials varied considerably. The aim of the present meta-analysis was to explore the robustness of the renal composite benefits using a uniform definition. (2) Methods: A web-based search was conducted using the Cochrane Library to identify the relevant articles for meta-analysis. RStudio (1 July 2022, Build 554) software was used to conduct the meta-analysis. Hazard ratio (HR) was the effect size used to estimate the renal composite benefit, and prediction interval was used to detect heterogeneity. In view of the differing baseline characteristic of the trials as well as different molecules used, a random effects model was used. (3) Results: There were 12 trials including 78,781 patients, identified using the search strategy, and a five-point Cochrane risk-of-bias was used to assess quality of the publications. In the overall estimation (irrespective of the definition used for the renal composite) the HR was 0.68 (95% CI 0.60–0.76, prediction interval: 0.48–0.95) in favour of SGLT-2is, devoid of heterogeneity. While using a uniform definition of eGFR ≥ 40%decline, ESKD, or renal death, the HR was 0.64 (95% CI 0.53–0.78); using eGFR ≥ 50%decline, ESKD, or renal death the HR was 0.75 (95% CI 0.59–0.97); and with doubling of serum creatinine, renal replacement therapy, or renal death, the HR was 0.67 (95% CI 0.55–0.83) in favour of SGLT-2is. However, significant heterogeneity was encountered with all these three definitions. (4) Conclusion: There is a need to analyse the renal outcomes using a uniform definition in future trials. The presence of heterogeneity might disappear with the pooling of larger number of trials. However, if heterogeneity persists, we need to identify other clinical or laboratory attributes (in addition to SGLT-2is) responsible for the positive renal outcomes.

## 1. Introduction

Sodium glucose co-transporter 2 inhibitors (SGLT-2is) have become the most prominent group of drugs used for slowing the progression of diabetic nephropathy [1]. The benefits of these drugs are evident based on their positive effect on renal composite end points (eGFR decline, requirement for renal replacement therapy, or renal death). In addition, SGLT-2is lead to a significant reduction in the progression of albuminuria [2]. As a result, guidelines recommend SGLT-2is as the first-line agent for type 2 diabetes (T2D) patients with established chronic kidney disease (CKD), defined as a sustained reduction in eGFR < 60 mL/min or urine albumin creatinine ratio (UACR) ≥ 200 mg/g [3]. However, there are significant differences in the ways that the renal composite outcome is defined across studies. The eGFR decline has been defined as a ≥40% decline, ≥50% decline, or a doubling of serum creatinine. The EMPAREG outcome trial demonstrated how an inadequate definition could lead to confusion. The inclusion of unexplained death as part of cardiovascular (CV) death led to several additional analyses (excluding unexplained deaths or a Bayesian analysis) to substantiate the original claim [4,5]. The beneficial effects of SGLT-2is on renal composite end points have been frequently analysed despite these definitional variations. The purpose of this meta-analysis was to explore whether analysing the renal composite end point using the different definitions used for eGFR decline yielded benefits consistent with the original claim.

The meta-analysis was conducted in accordance with the PICO question format:

P (patient population) = patients diagnosed with T2D.
I (intervention) = received drugs belonging to the SGLT-2i group.
C (control group) = compared to placebo.
O (outcome) = beneficial effects of SGLT-2is on renal composite outcomes compared with placebo irrespective of the definitions used, followed by the reanalysis of the data using the different definitions used to describe the eGFR decline aspect of the renal composite outcomes.

## 2. Materials and Methods

This review was performed in accordance with the Preferred Reporting Items for Systematic Reviews and Meta-Analyses (PRISMA) statement [6]. Our review protocol was prospectively registered [7].

### 2.1. Search Strategy and Eligibility Criteria

**Figure 1:**
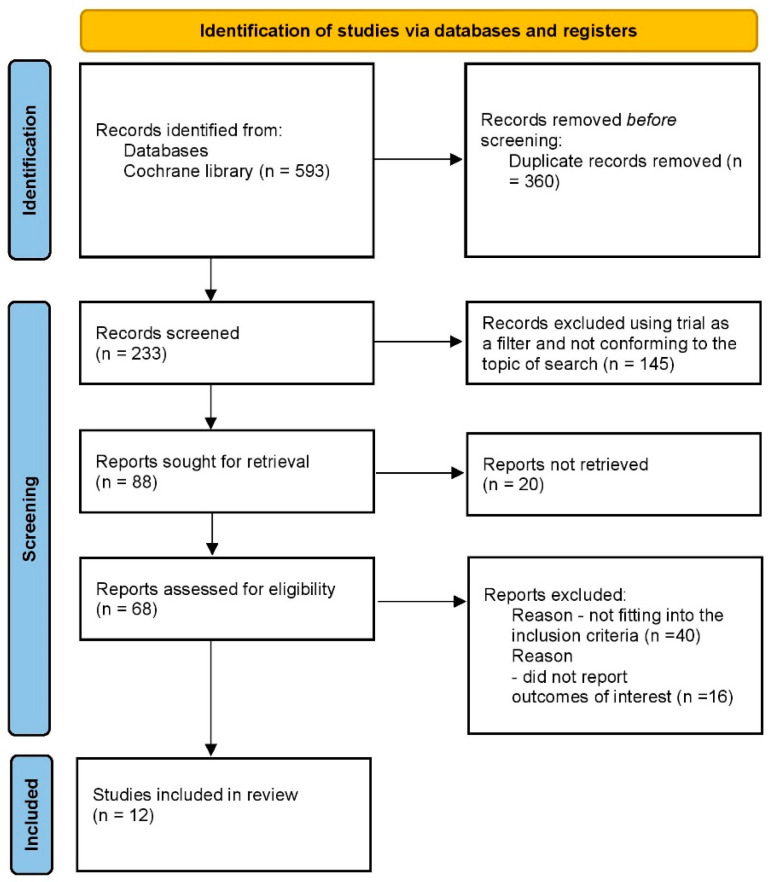
Study selection process.

The Cochrane Library electronic database was searched by the authors (S.G. and A.G.) without any date or language restrictions. The authors also manually searched for abstracts related to the topic. The search keywords included the following terms: ((“Type 2 diabetes mellitus”{MeSH}, OR “T2DM”) OR (“Sodium glucose co-transporter 2 inhibitors” OR “SGLT-2 inhibitors” OR “Empagliflozin” OR “Dapagliflozin” OR “Canagliflozin” OR “Ertugliflozin” OR “Ipragliflozin”)) AND (“Renal composite” OR “Kidney outcomes”). The full search strategy can be found in Figure 1 and the Supplementary Material S1.

### 2.2. Study Selection and Eligibility Criteria

After performing the initial web search, the final studies included for analysis were determined based on the eligibility criteria specified in the protocol. The eligibility criteria comprised phase 3 randomized controlled trials (RCTs) with the characteristics detailed below:

- T2D.
- Age: more than or equal to 18 years.
- Comparator arm: placebo.
- Clear mention of the renal composite outcomes, including the individual components in the selected citations.

The exclusion criteria were as follows:

- Non-T2D patients (type 1 diabetes, gestational diabetes, type 3c diabetes, etc.).
- Acute or decompensated disorder pertaining to any organ.
- Non-RCTs.
- Less than eighteen years of age.
- The comparator arm including agents capable of altering the primary outcome.

### 2.3. Data Extraction

The authors (S.G. and A.G.) independently conducted the search and then compared their data. Those articles which were either duplicate or did not conform to the eligibility criteria were excluded. Any disagreements between the authors were resolved via mutual agreement, with SG1 performing an independent examination of the results.

### 2.4. Risk of Bias

Biases associated with the individual studies was assessed using the Cochrane Collaboration Risk of Bias 2.0 tool (Supplementary Material S2). The authors evaluated the individual articles, and any disagreements were tackled via consensus. The assessment of publication bias was conducted qualitatively (when citations were less than 10) and quantitatively using Egger’s regression test for the overall analysis. A qualitative analysis including assessment for outliers was conducted for the subgroups since there were fewer than ten citations (Supplementary Material S3).

### 2.5. Statistical Analysis

The meta-analysis was conducted in two steps.

Step 1: An overall analysis was performed to determine the beneficial effects of SGLT-2is on renal composite outcomes, irrespective of the baseline definition. The hazard ratio (HR) was selected as the effect size, and the precision of the estimation was assessed using a 95% confidence interval. The random effects model was used to conduct the meta-analysis. Heterogeneity was assessed using the prediction interval.

Step 2: The meta-analysis was repeated using the three most frequently used definitions for eGFR decline (≥40% GFR decline, ≥50% sustained decline in eGFR, and the doubling of serum creatinine). The effect size (HR) and prediction interval were assessed for each of these end points. The RStudio version (1 July 2022, Build 554).was used to conduct the meta-analysis and assess funnel plots. The online Cochrane risk of bias tool was used to assess and construct figures describing biases associated with individual studies. The codes used for conducting the meta-analysis are provided in the Supplementary Material S4.

## 3. Results

### 3.1. Baseline Characteristics and Risk of Bias

Ultimately, there were 12 citations used in the meta-analysis [8,9,10,11,12,13,14,15,16,17,18,19]. A total of 78,781 patients were included in this analysis, with 42,658 in the SGLT-2i arm and 36,123 in the comparison arm (Table 1). The mean age was between 63 years and 71.8 years. The definition of the primary outcome and the way eGFR decline was described was not uniform (Table 1). The mean baseline eGFR ranged from >60 mL/min to 12.2 mL/min.

In the DECLARE TIMI-58 RCT, there were some concerns related to deviations from intended interventions and missing data, and there was a high level of concern related to selection of the reported results. There were some concerns related to outcome reporting and the selection of reported results in the CANVAS trial. Since HR was used as the measure of effect size, Egger’s regression test was conducted to detect funnel plot asymmetry. The uniform definition subsets had fewer than 10 studies; hence, a qualitative assessment along with an assessment of outliers was conducted (Supplementary Material S3). There was no evidence of funnel plot asymmetry in the overall data, and no outliers were detected in the uniform definition subsets.

### 3.2. Renal Composite Outcomes Irrespective of Baseline Definition

**Figure 2:**
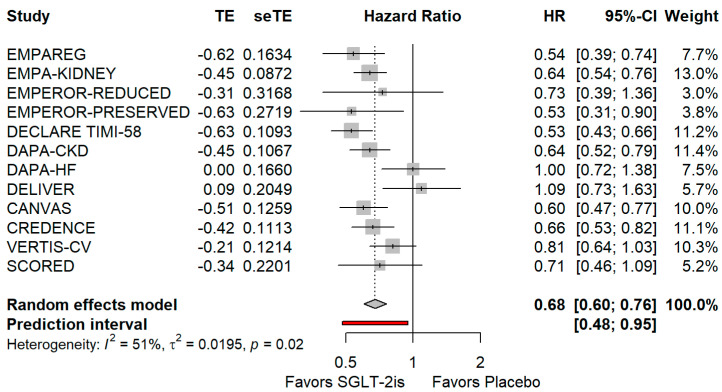
Renal composite benefits with SGLT-2is compared to the placebo, irrespective of baseline definition for eGFR decline [8,9,10,11,12,13,14,15,16,17,18,19]. TE: logHR; seTE: standard error of logHR. The red strip indicates prediction interval.

There was a 32% lower risk of progression of the renal composite in the SGLT-2i arm compared to the placebo group (95% CI 0.60–0.76) (Figure 2). A prediction interval of 0.48–0.95 indicated a lack of heterogeneity. 

### 3.3. Uniform Definition 1: Preventing ≥ 40% GFR Decline in eGFR

**Figure 3:**
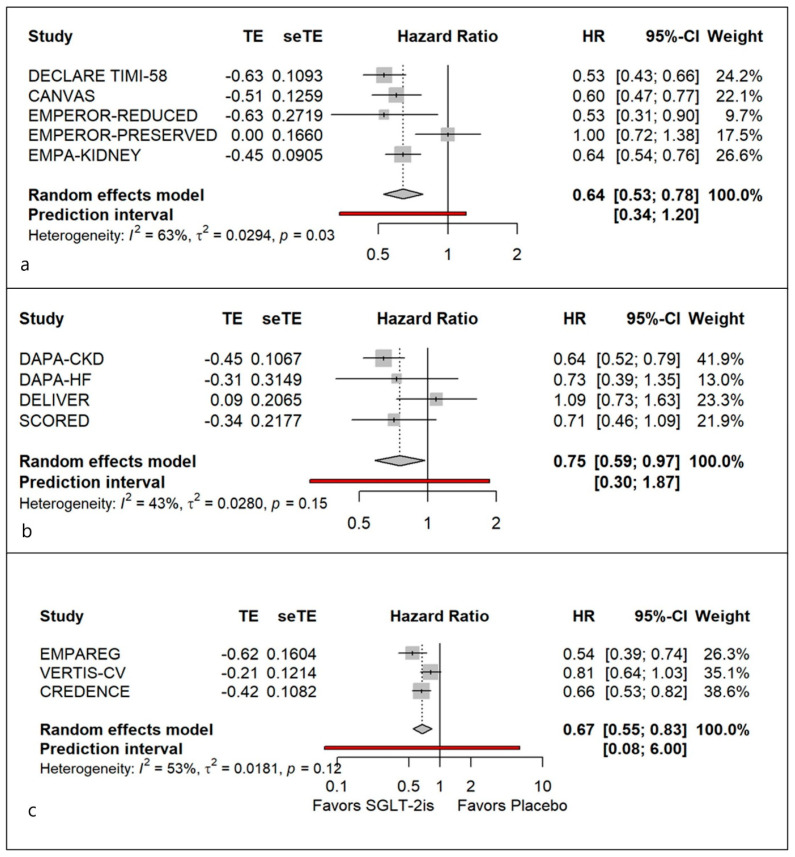
Renal composite benefits with SGLT-2is compared to placebo depending on the specific eGFR decline definition used in the renal composite [8,9,10,11,12,13,14,15,16,17,18,19]: (**a**) ≥40% GFR decline in eGFR; (**b**) ≥50% GFR decline in eGFR; (**c**) doubling of serum creatinine. The red strip indicates prediction interval.

When the renal composite included ≥40% GFR decline in eGFR, there was a 36% lower risk of progression in the SGLT-2i group than in the placebo group (95% CI 0.53–0.78). However, the prediction interval indicated that the result was not generalizable to a wider population (PI 0.34–1.20), with a 20% increased risk of worsening of renal composite in certain populations (Figure 3a).

### 3.4. Uniform Definition 2: Preventing a ≥ 50% GFR Decline in eGFR

When the renal composite included ≥50% GFR decline in eGFR, there was a 25% lower risk of progression in the SGLT-2i group than in the placebo group (95% CI 0.59–0.97). However, the prediction interval indicated that the result was not generalizable to a wider population (PI 0.30–1.87). There are populations wherein a 70% risk reduction can be expected (superresponders), in contrast to populations where we might encounter an 87% risk for worsening of the renal composite (Figure 3b). The dispersion of the true effect size was very large when employing universal Definition 2.

### 3.5. Uniform Definition 3: Preventing Doubling of Serum Creatinine

When the renal composite included doubling of serum creatinine, there was a 33% lower risk of progression in the SGLT-2i group than in the placebo group. The positive outcome finding was offset by a large prediction interval (0.08–6.00), indicating that this result was not generalizable (Figure 3c).

## 4. Discussion

The management of T2D changes frequently with the availability of data from organ-specific outcome trials. The availability of agents (SGLT-2is and glucagon-like peptide-1 receptor agonists (GLP1-RA)) with target organ protection attributes has brought about dramatic changes in the way guidelines are prepared. In contrast to the traditional gluco-centric approach, the focus of modern consensus statements and guidelines is to introduce agents with target organ protection early in the disease process alongside other agents targeting metabolic components. The 2023 American Diabetes Association consensus statement recommends SGLT-2is or GLP1-RA as a first-line agent (above metformin) in T2D patients with established or high cardio-renal risks [20]. SGLT-2is have become the cornerstone of therapy in the management of chronic renal disease and heart failure. From the renal perspective, SGLT-2is are considered first-line agents. Robust data from dedicated renal outcome trials (DAPA-CKD and EMPA-Kidney) led to guidelines making these recommendations [8,15]. However, there are various definitions of the primary end-point (renal composite) across the trials, and this heterogeneity needs to be analysed in detail.

### 4.1. Literature Review

The renal composite benefits associated with SGLT-2is have been documented in the DAPA-CKD and EMPA-kidney trials. There was a similar 36% reduction in primary outcomes (renal composite) compared to placebo in those with T2D at baseline in both trials [8,15]. Meta-analyses exploring the renal composite benefits of SGLT-2is in T2D have been uniform in their conclusions. Giugliano et al. documented a 35% reduction in the risk of the renal composite with SGLT-2is (95% CI 0.56–0.75) in patients irrespective of baseline T2D by analysing eleven RCTs [21]. Another meta-analysis of 13 RCTs reported a 38% reduction in the relative risk of the renal composite in the SGLT-2is group among patients with T2D (95% CI 0.56–0.68), with the greatest benefits observed for slowing the progression of kidney disease in patients with relatively preserved eGFR [22].

One of the important components of the renal composite is the proportion of eGFR decline. Hence, it is of paramount importance that there is a standardised definition. This is especially important when conducting a meta-analysis since the inclusion of various definitions might result in skewed summary effect sizes. The EMPA KIDNEY, EMPEROR PRESERVED & REDUCED, DECLARE TIMI-58, and CANVAS trials used ≥ 40% eGFR decline as a component of the renal composite; the DAPA HF, DELIVER, DAPA CKD, and SCORED trials used ≥ 50% eGFR decline as the outcome; and the EMPA REG, CREDENCE, VERTIS-CV used the doubling of serum creatinine as the outcome. None of these differences were considered while assessing the summary effect size in meta-analyses. In addition, the heterogeneity of the effect size was erroneously assessed by using I^2^ statistics in the meta-analyses instead of the prediction interval [23].

### 4.2. Findings from This Meta-Analysis

The 32% reduction in the HR of the renal composite in patients with T2D, irrespective of the baseline definition, was consistent with most previously reported meta-analyses. However, we assessed the dispersion of the true effect size using the prediction interval (measure of heterogeneity) in contrast to I^2^ statistics. The prediction interval ranged between 0.48 and 0.95, indicating that the finding was generalizable. However, analysing the data using a common eGFR definition resulted in a lack of generalizability of the data. Using an eGFR decline definition of ≥40%, the HR was 0.64 (95% CI 0.53–0.78), while the prediction interval ranged between 0.34–1.20, indicating a lack of generalizability. A very similar trend was observed when the outcomes were an eGFR decline of ≥50% (HR 0.75, 95% CI 0.59–0.97; PI 0.30–1.87) and doubling of serum creatinine (HR 0.67, 95% CI 0.55–0.83; PI 0.08–6.00).

### 4.3. Limitations and Strengths

There were several limitations associated with this meta-analysis. The analysis was conducted using published outcomes and did not use individual patient data. Second, a quantitative assessment of funnel plot asymmetry could not be performed on the subgroups as the number of studies were too small. Hence, a certain degree of selection bias is expected to be present. Third, the prediction interval assessing heterogeneity can be convinsingly assessed in a meta-analysis including at least ten studies. Although the overall analysis was carried out with 13 studies, the subgroups were analysed with fewer than 10 citations. Hence, the assessment of heterogeneity might not be as robust as the pooled estimate.

Pooling the relevant citations resulted in analysis of large number of patients, which was the main strength of this analysis. In addition, approaching the issue from a data heterogeneity point of view helped us identify areas of future research to identify the sources of heterogeneity. Although having at least ten studies can increase the accuracy of a prediction interval, a minimum of three studies can give us a fairly good idea about the dispersion of the true effect size.

### 4.4. Research Recommendations

Based on the findings of this meta-analysis, the following research recommendations can be made:

- It is necessary to standardize the definition of renal composite end points.
- To develop a standardized definition of renal composite benefits of SGLT-2is in T2D patients, more data need to be accumulated with a uniform definition.
- In view of the heterogeneity detected (leading to a lack of generalizability) with all three definitions of eGFR decline, additional studies are required to identify clinical and biochemical attributes that can be used to identify responders and non-responders to SGLT-2is.

## 5. Conclusions

SGLT-2is have become the first-line therapy for preventing and slowing the progression of chronic kidney disease in T2D patients. However, the lack of a uniform definition and significant heterogeneity associated with the individual definitions must be considered before generalizing the findings regarding the effects of SGLT-2is. The sources of heterogeneity must be considered in future trials.

## Figures and Tables

**Table 1:** Baseline characteristics of the studies included in the meta-analysis.

Study	Year	Intervention	Intervention/Placebo (n)	Mean Age	Renal Composite	Baseline eGFR (mL/min)
EMPA KIDNEY [8]	2022	Empagliflozin	1525/1515	63.9 ± 13.9	End-stage kidney disease, a sustained estimated glomerular filtration rate (eGFR) < 10 mL/min/1.73 m^2^, renal death, or a sustained decline of ≥40% in eGFR from randomisation.	37.4 ± 14.5 (mean)
EMPAREG [9]	2015	Empagliflozin	4645/2323	63.0 ± 8.6	Doubling of serum creatinine, initiation of renal replacement therapy, or death due to renal disease	≥60 (74.1%), 45 to <60 (17.8%), <45 (8.1%)
EMPEROR PRESERVED [10]	2021	Empagliflozin	1466/1472	70.9 ± 9.0	Chronic dialysis, renal transplantation, sustained reduction of ≥40% in estimated GFR	59.7 ± 20.7 (Mean)
EMPEROR REDUCED [11]	2022	Empagliflozin	927/929	67.6 ± 11.6	Chronic dialysis, kidney transplant, sustained reduction of ≥40% eGFR, or sustained eGFR < 15 mL/min/1.73 m^2^ if eGFR was >30 mL/min/1.73 m^2^ or <10 mL/min/1.73 m^2^ for patients with baseline eGFR ≤ 30 mL/min/1.73 m^2^	62.7 ± 21.1 (Mean)
DECLARE TIMI-58 [12]	2019	Dapagliflozin	8582/8578	63.9 + 6.8	A 40% decrease in eGFR, ESRD, or renal death	85.4 ± 15.8
DAPA-HF [13]	2019	Dapagliflozin	2139/2139	66.2 + 11.0	A reduction of 50% or more in the estimated GFR sustained for at least 28 days, end-stage renal disease, or death from renal causes.	66.0 ± 19.6 (Mean)
DELIVER [14]	2022	Dapagliflozin	3131/3131	71.8 ± 9.6	Composite of decline in estimated GFR of ≥50%, end-stage kidney disease, or death from renal causes	61 ± 19 (Mean)
DAPA-CKD [15]	2020	Dapagliflozin	1455/1451	61.8 ± 12.1	Composite of decline in estimated GFR of ≥50%, end-stage kidney disease, or death from renal causes	43.2 ± 12.3 (Mean)
CANVAS [16]	2017	Canagliflozin	5795/4347	63.2 + 8 3	A 40% decrease in eGFR, renal death or renal replacement therapy requirement	76.7 ± 20.3 (Mean)
CREDENCE [17]	2019	Canagliflozin	2202/2199	62.9 ± 9.2	End stage kidney disease, doubling of serum creatinine, renal death	56.3 ± 18.2 (Mean)
VERTIS-CV [18]	2021	Ertugliflozin	5499/2747	64.4 ± 8.1	Doubling creatinine, ESKD, renal death	76.1 ± 20.9 (Mean)
SCORED [19]	2021	Sotagliflozin	5292/5292	69 (63–74) [Median–IQR]	A ≥50% sustained decline eGFR or end-stage renal disease or renal death	44.4 (37.0–51.3) [Median–IQR]

## Data Availability

All data are available in the manuscript and Supplementary Material provided.

## Supplementary Materials

The following supporting information can be downloaded at: https://www.mdpi.com/article/10.3390/jcm12206462/s1. S1: Cochrane library search strategy. S2: Cochrane risk of bias. S3: Assessment of funnel plot asymmetry. S4: Codes used to conduct the meta-analysis.
