# Supplementary material for: The Renal Composite Benefit of Sodium Glucose Co-Transporter 2 Inhibitors Should Ideally Be Assessed Based on a Standardised Definition: A Meta-Analysis of Randomised Controlled Trials
Source: J Clin Med. 2023 Oct 11;12(20):6462. doi: 10.3390/jcm12206462 (PMC10607004; doi:10.3390/jcm12206462)
Supplement: Supplementary file 1 [file jcm-12-06462-s001.zip › jcm-2633821-supplementary.pdf]

Supplementary Materials

[S1]. Cochrane library search strategy:

Search Name: Flozins and renal composite

Date Run: 14/06/2023 21:12:55

Comment:

ID	Search	Hits
#1	MeSH descriptor: [Diabetes Mellitus, Type 2] explode all trees	23067
#2	T2D	4590
#3	MeSH descriptor: [Sodium-Glucose Transport Proteins] explode all trees	169
#4	sglt-2 inhibitors	248
#5	Empagliflozin	1693
#6	Dapagliflozin	1810
#7	Canagliflozin	761
#8	Ertugliflozin	200
#9	Sotagliflozin	141
#10	Ipragliflozin	170
#11	Placebo	374993
#12	Renal composite	3295
#13	Kidney outcomes	13014
#14	Doubling of serum creatinine	574
#15	#1 OR #2	26058
#16	#3 OR #4 OR #5 OR #6 OR #7 OR #8 OR #9 OR #10 OR #11	376875
#17	#12 OR #13 OR #14	15291
#18	#15 AND #16 AND #17	593

[S2]. Cochrane risk of bias:

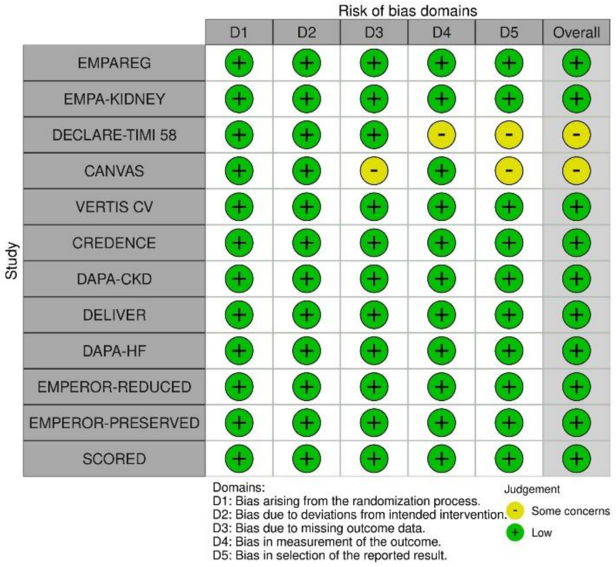

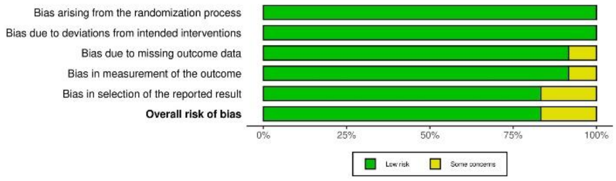

[S3]. Assessment of funnel plot asymmetry:

1. Overall data (quantitative assessment):

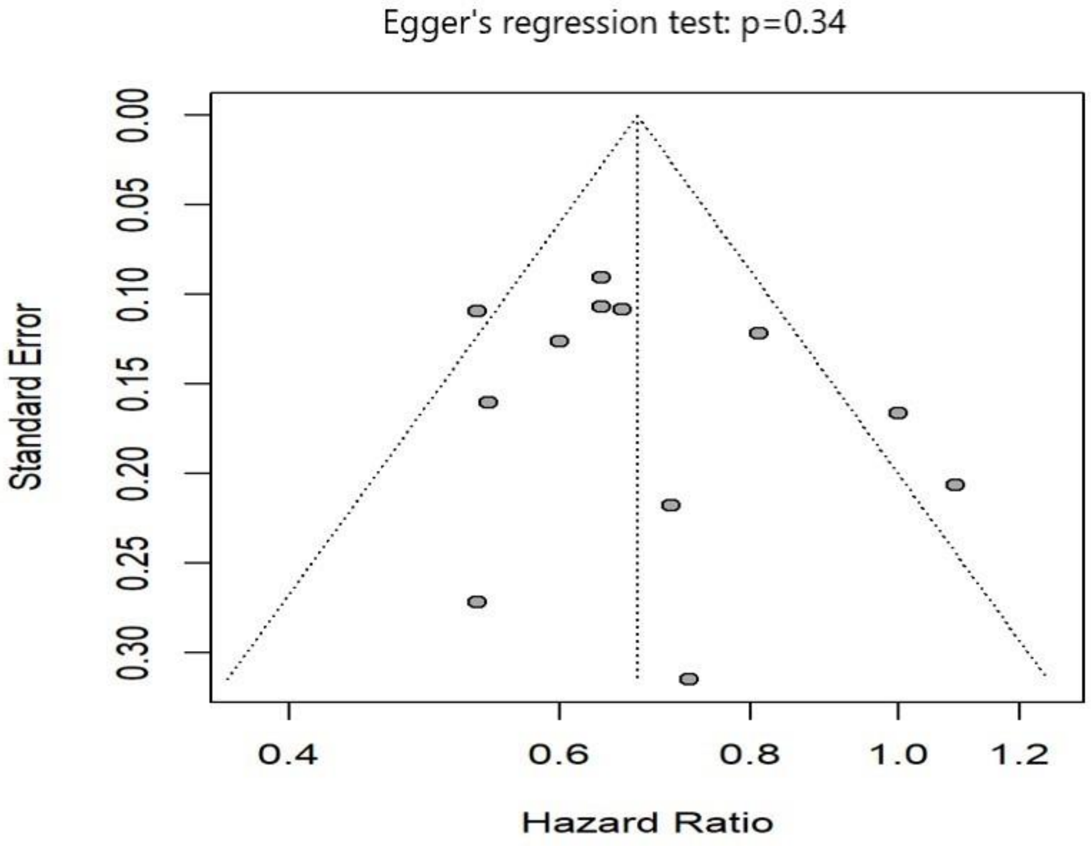

Linear regression test of funnel plot asymmetry

Test result: t = 0.98, df = 10, p-value = 0.3485

Sample estimates:

bias	se.bias	intercept	se.intercept
1.2205	1.2410	-0.5690	0.1682

Details:

- multiplicative residual heterogeneity variance (tau^2 = 2.0282)
- predictor: standard error
- weight: inverse variance
- reference: Egger et al. (1997), BMJ

No outliers detected (random-effects model).

2. Uniform definition 1: ≥40% GFR decline (Qualitative assessment)

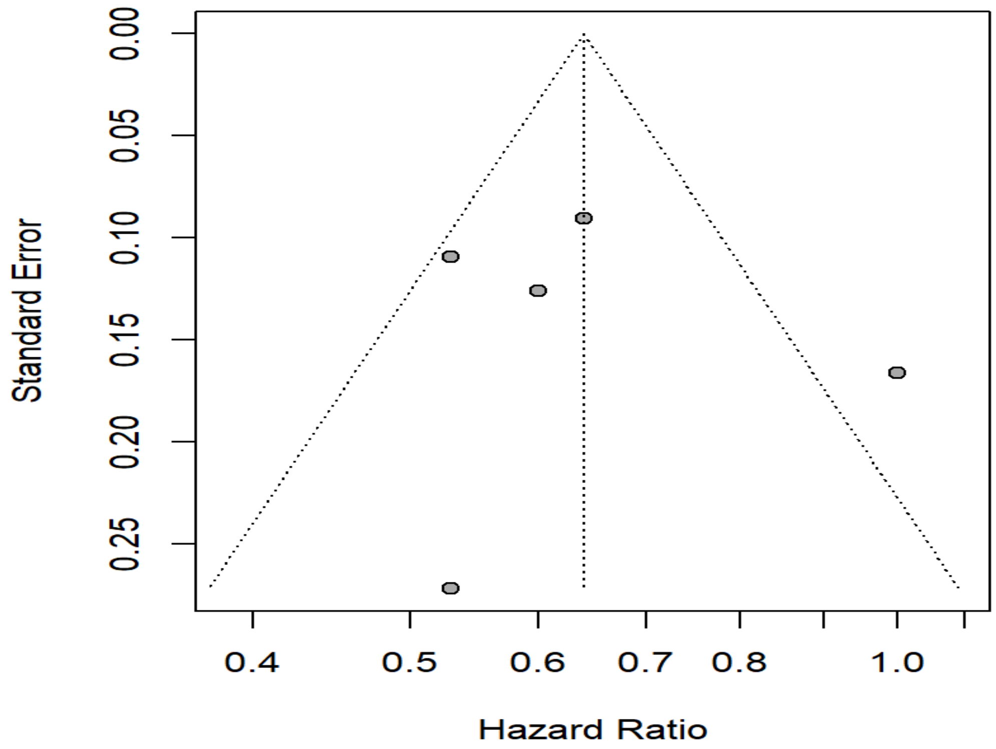

No outliers detected (random-effects model).

3. Uniform definition 2: ≥50% GFR decline (Qualitative assessment)

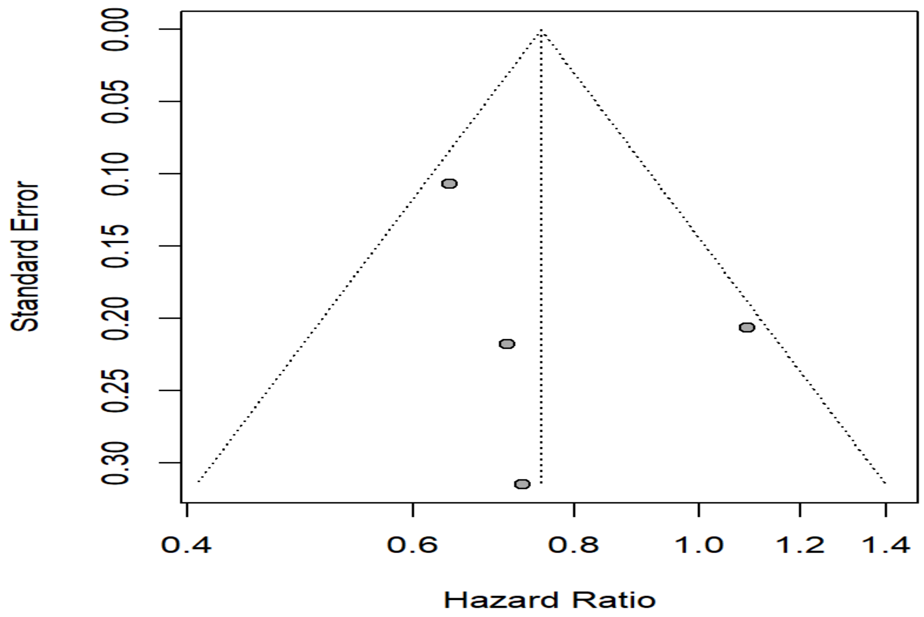

No outliers detected (random-effects model).

4. Uniform definition 3: Doubling of serum creatinine

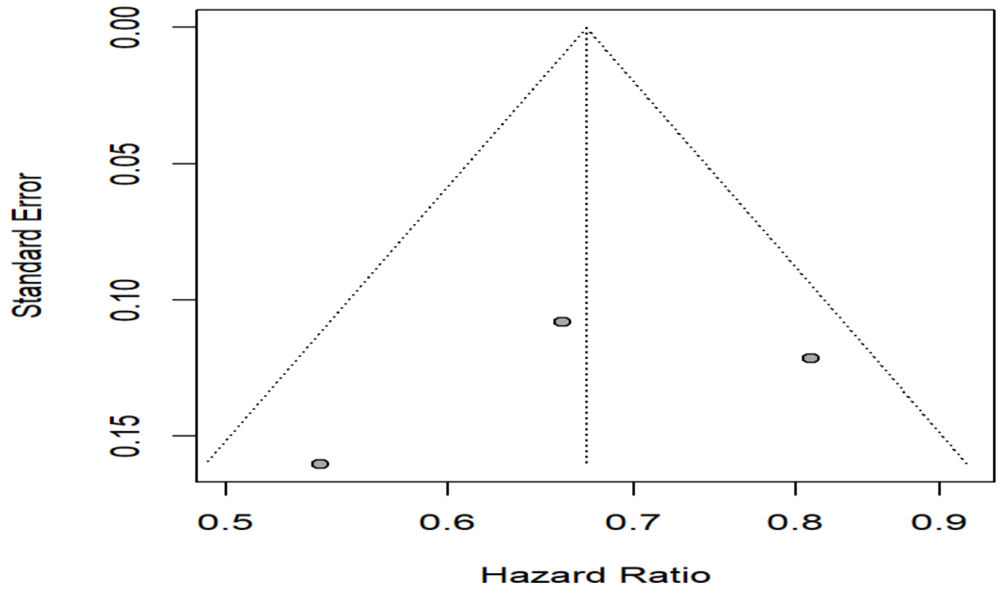

No outliers detected (random-effects model).

[S4]. Codes used to conduct the meta-analysis:

1. The meta-analysis:

library(dmetar)

```
library(meta)
study <- c("EMPAREG", "EMPA-KIDNEY","EMPEROR-REDUCED", "EMPEROR-PRESERVED",
      "DECLARE TIMI-58","DAPA-CKD", "DAPA-HF", "DELIVER", "CANVAS", "CREDENCE",
      "VERTIS-CV", "SCORED")
HR <- c(0.54,0.64,0.73,0.53,0.53,0.64,1.00,1.09,0.60,0.66,0.81,0.71)
lower.HR <- c(0.39,0.54,0.39,0.31,0.43,0.52,0.72,0.73,0.47,0.53,0.64,0.46)
upper.HR <- c(0.74,0.76,1.35,0.90,0.66,0.79,1.38,1.63,0.77,0.82,1.03,1.09)
m1gen <- metagen(log(HR), lower = log(lower.HR), upper = log(upper.HR),
          studlab = study,
          comb.fixed = FALSE,
          comb.random = TRUE,
          method.tau = "DL",
          hakn = FALSE,
          prediction = TRUE,
          sm = "HR")
m1gen
forest(m1gen)
forest(m1gen, xlab = "Favors SGLT-2is      Favors Placebo", showweights = TRUE)
```

#Uniform definition 1

```
study <- c("DECLARE TIMI-58", "CANVAS", "EMPEROR-REDUCED", "EMPEROR-PRESERVED",
      "EMPA-KIDNEY")
HR <- c(0.53, 0.60, 0.53, 1.00,0.64)
lower.HR <- c(0.43, 0.47, 0.31, 0.72,0.54)
upper.HR <- c(0.66, 0.77, 0.90, 1.38,0.77)
m2gen <- metagen(log(HR), lower = log(lower.HR), upper = log(upper.HR),
          studlab = study,
          comb.fixed = FALSE,
          comb.random = TRUE,
          method.tau = "DL",
          hakn = FALSE,
```

```
              prediction = TRUE,
              sm = "HR")
m2gen
forest(m2gen)
forest(m2gen, xlab = "Favors SGLT-2is      Favors Placebo", showweights = TRUE)
```

#Uniform definition 2

```
study <- c("DAPA-CKD", "DAPA-HF", "DELIVER", "SCORED")
HR <- c(0.64, 0.73, 1.09,0.71)
lower.HR <- c(0.52, 0.39, 0.73,0.46)
upper.HR <- c(0.79, 1.34, 1.64,1.08)
m3gen <- metagen(log(HR), lower = log(lower.HR), upper = log(upper.HR),
              studlab = study,
              comb.fixed = FALSE,
              comb.random = TRUE,
              method.tau = "DL",
              hakn = FALSE,
              prediction = TRUE,
              sm = "HR")
forest(m3gen)
forest(m3gen, xlab = "Favors SGLT-2is      Favors Placebo", showweights = TRUE)
```

#Uniform definition 3

```
study <- c("EMPAREG", "VERTIS-CV", "CREDENCE")
HR <- c(0.54,0.81,0.66)
lower.HR <- c(0.4,0.64,0.53)
upper.HR <- c(0.75,1.03,0.81)
m4gen <- metagen(log(HR), lower = log(lower.HR), upper = log(upper.HR),
              studlab = study,
              comb.fixed = FALSE,
              comb.random = TRUE,
```

```
            method.tau = "DL",
            hakn = FALSE,
            prediction = TRUE,
            sm = "HR")
forest(m4gen)
forest(m4gen, xlab = "Favors SGLT-2is     Favors Placebo", showweights = TRUE)
```

2. The funnel plot asymmetry:

a. Overall:

```
library(dmetar)
library(meta)
study <- c("EMPAREG", "DECLARE TIMI-58","CANVAS", "VERTIS-CV", "CREDENCE", "DAPA-CKD",
       "EMPEROR-REDUCED", "EMPEROR-PRESERVED", "DAPA-HF", "SCORED", "EMPA-KIDNEY",
       "DELIVER")
HR <- c(0.54,0.53,0.60,0.81,0.66,0.64,0.73,0.53,1.00,0.71,0.64,1.09)
lower.HR <- c(0.4,0.43,0.47,0.64,0.53,0.52,0.39,0.31,0.72,0.46,0.54,0.73)
upper.HR <- c(0.75,0.66,0.77,1.03,0.81,0.79,1.34,0.90,1.38,1.08,0.77,1.64)
m1gen <- metagen(log(HR), lower = log(lower.HR), upper = log(upper.HR),
            studlab = study,
            comb.fixed = FALSE,
            comb.random = TRUE,
            method.tau = "DL",
            hakn = FALSE,
            prediction = TRUE,
            sm = "HR")
m1gen
funnel(m1gen)
find.outliers(m1gen)
metabias(m1gen, method.bias = "linreg")
```

b. Uniform definition 1: ≥40% GFR decline.

```
study <- c("DECLARE TIMI-58", "CANVAS", "EMPEROR-REDUCED", "EMPEROR-PRESERVED",
      "EMPA-KIDNEY")
HR <- c(0.53, 0.60, 0.53, 1.00,0.64)
lower.HR <- c(0.43, 0.47, 0.31, 0.72,0.54)
upper.HR <- c(0.66, 0.77, 0.90, 1.38,0.77)
m2gen <- metagen(log(HR), lower = log(lower.HR), upper = log(upper.HR),
          studlab = study,
          comb.fixed = FALSE,
          comb.random = TRUE,
          method.tau = "DL",
          hakn = FALSE,
          prediction = TRUE,
          sm = "HR")
m2gen
find.outliers(m2gen)
funnel(m2gen)
```

c. Uniform definition 2: ≥50% GFR decline:

```
study <- c("DAPA-CKD", "DAPA-HF", "SCORED", "DELIVER")
HR <- c(0.64, 0.73, 0.71,1.09)
lower.HR <- c(0.52, 0.39, 0.46,0.73)
upper.HR <- c(0.79, 1.34, 1.08,1.64)
m3gen <- metagen(log(HR), lower = log(lower.HR), upper = log(upper.HR),
          studlab = study,
          comb.fixed = FALSE,
          comb.random = TRUE,
          method.tau = "DL",
          hakn = FALSE,
          prediction = TRUE,
          sm = "HR")
```

```
find.outliers(m3gen)
funnel(m3gen)
```

d. Uniform definition 3: Doubling of serum creatinine.

```
study <- c("EMPAREG", "VERTIS-CV", "CREDENCE")
HR <- c(0.54,0.81,0.66)
lower.HR <- c(0.4,0.64,0.53)
upper.HR <- c(0.75,1.03,0.81)
m1gen <- metagen(log(HR), lower = log(lower.HR), upper = log(upper.HR),
          studlab = study,
          comb.fixed = FALSE,
          comb.random = TRUE,
          method.tau = "DL",
          hakn = FALSE,
          prediction = TRUE,
          sm = "HR")
m1gen
funnel(m1gen)
find.outliers(m1gen)
metabias(m1gen, method.bias = "linreg")
```
